# Supplementary figures and images for: Grad-seq analysis of Enterococcus faecalis and Enterococcus faecium provides a global view of RNA and protein complexes in these two opportunistic pathogens
Source: Microlife. 2022 Dec 27;4:uqac027. doi: 10.1093/femsml/uqac027 (PMC10117718; doi:10.1093/femsml/uqac027)

Figure S4

A

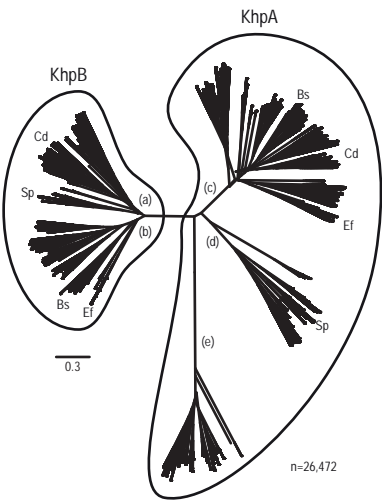

B

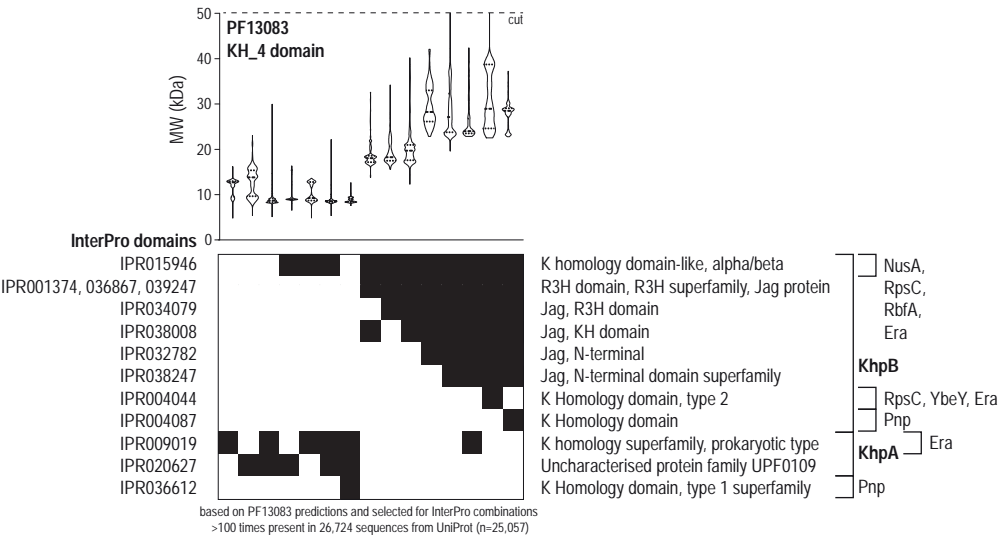

C

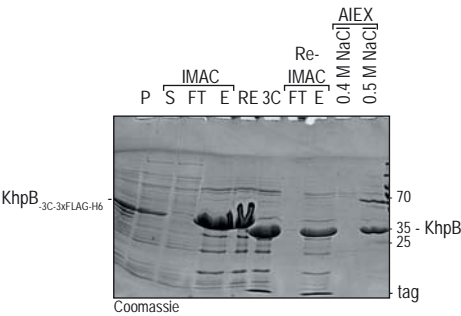

D

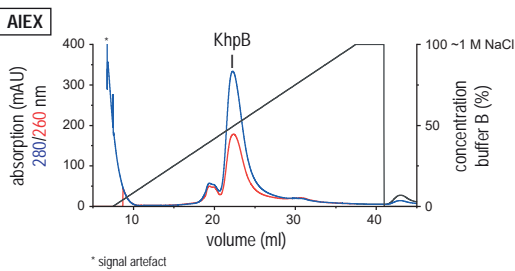

Supplement: uqac027_Supplemental_Files [file uqac027_supplemental_files.zip › FigS4.pdf]

Figure S1

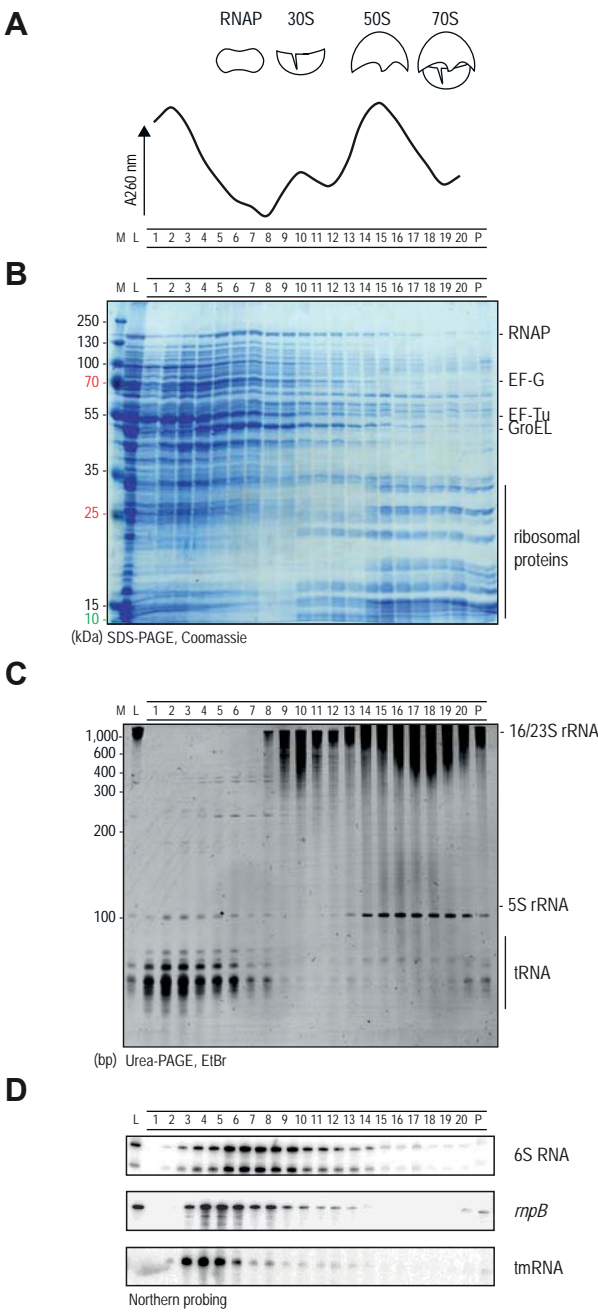

Supplement: uqac027_Supplemental_Files [file uqac027_supplemental_files.zip › FigS1.pdf]

Figure S2

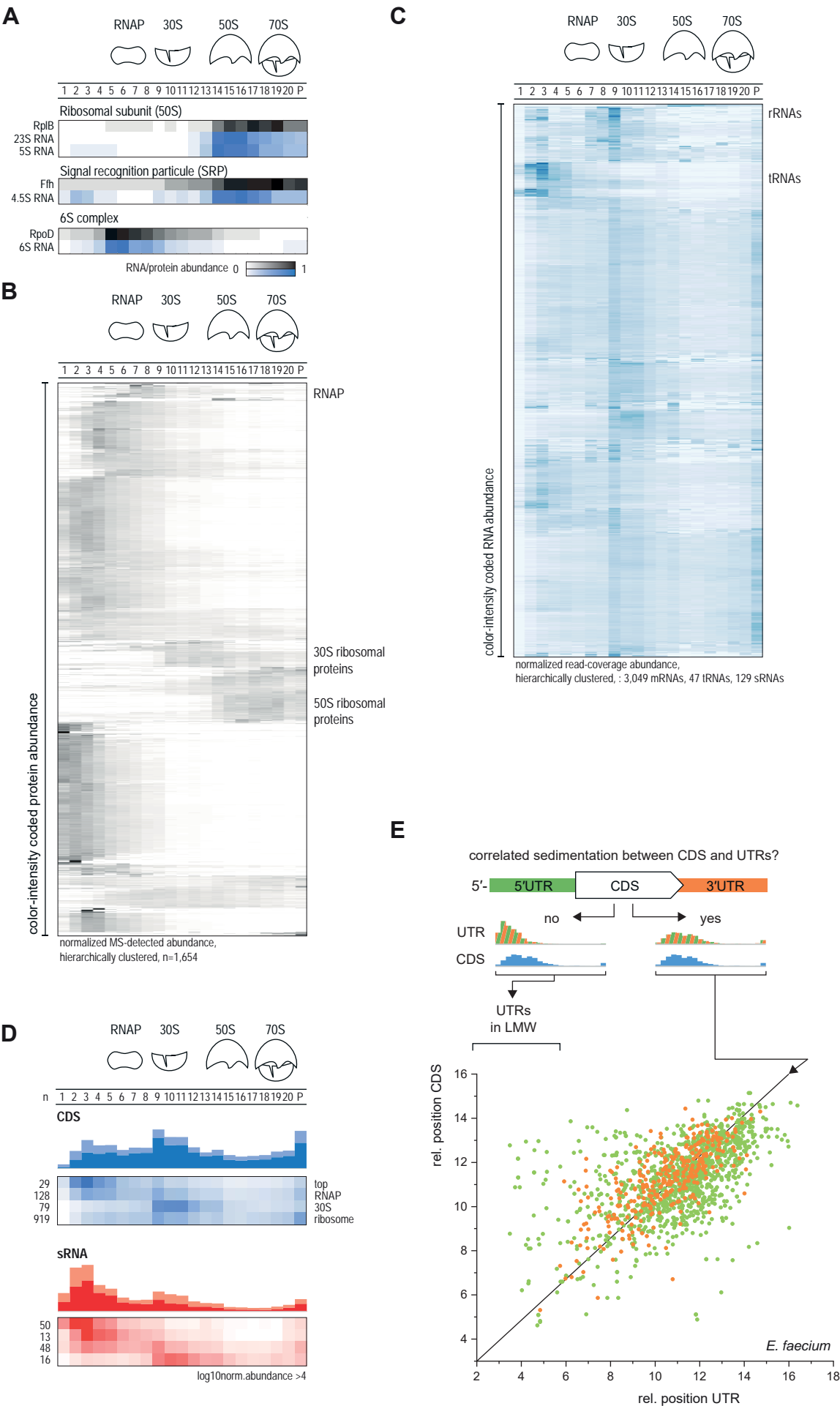

Supplement: uqac027_Supplemental_Files [file uqac027_supplemental_files.zip › FigS2.pdf]

Figure S3

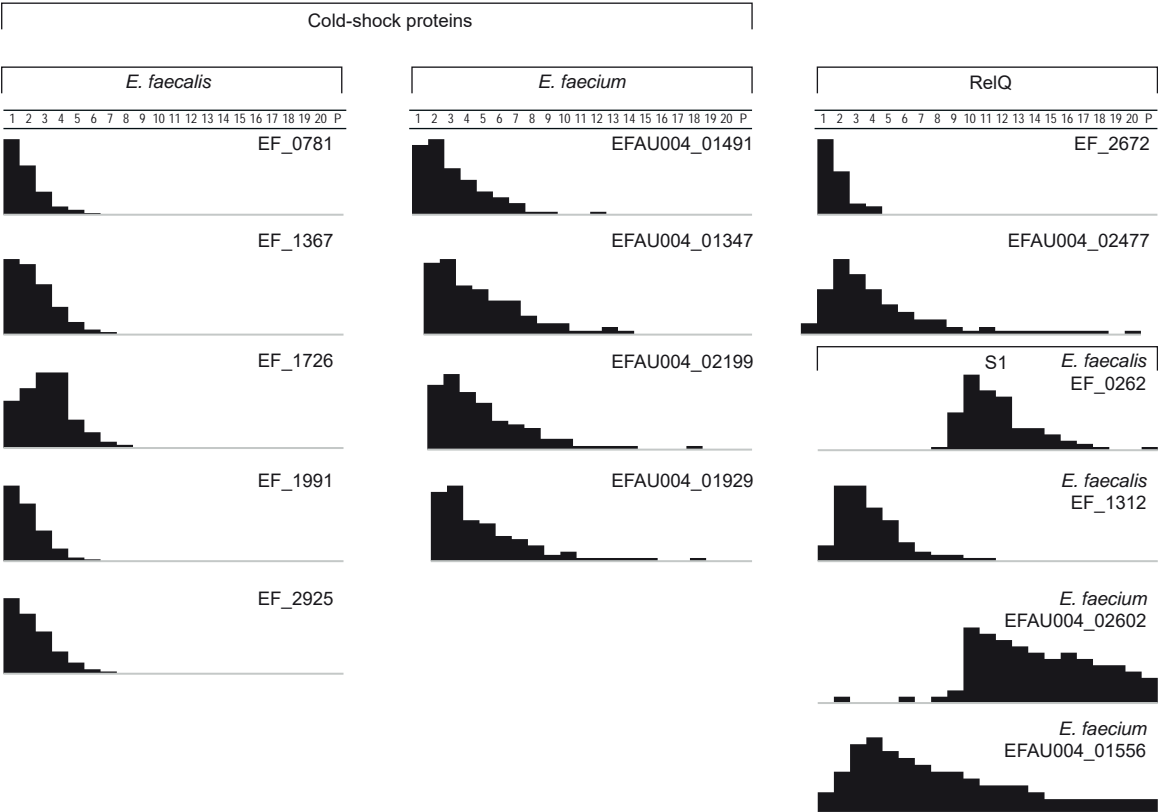

Supplement: uqac027_Supplemental_Files [file uqac027_supplemental_files.zip › FigS3.pdf]
